# Properties and Curing Kinetics of a Processable Binary Benzoxazine Blend

**DOI:** 10.1021/acsapm.3c02221

**Published:** 2023-11-15

**Authors:** Yue Tang, Henry E. Symons, Pierangelo Gobbo, Jeroen Sebastiaan Van Duijneveldt, Ian Hamerton, Sébastien Rochat

**Affiliations:** † School of Chemistry, University of Bristol, Cantock’s Close, Bristol BS8 1TS, U.K.; ‡ Department of Chemical and Pharmaceutical Sciences, University of Trieste, Via Giorgieri 1, Trieste 34127, Italy; § Bristol Composites Institute, School of Civil, Aerospace, and Design Engineering, University of Bristol, Queen’s Building, University Walk, Bristol BS8 1TR, U.K.; ∥ School of Engineering Mathematics and Technology, University of Bristol, Ada Lovelace Building, Tankard’s Close, Bristol BS8 1TW, U.K.

**Keywords:** benzoxazine, kinetics, processing, thermal properties, mechanical Properties

## Abstract

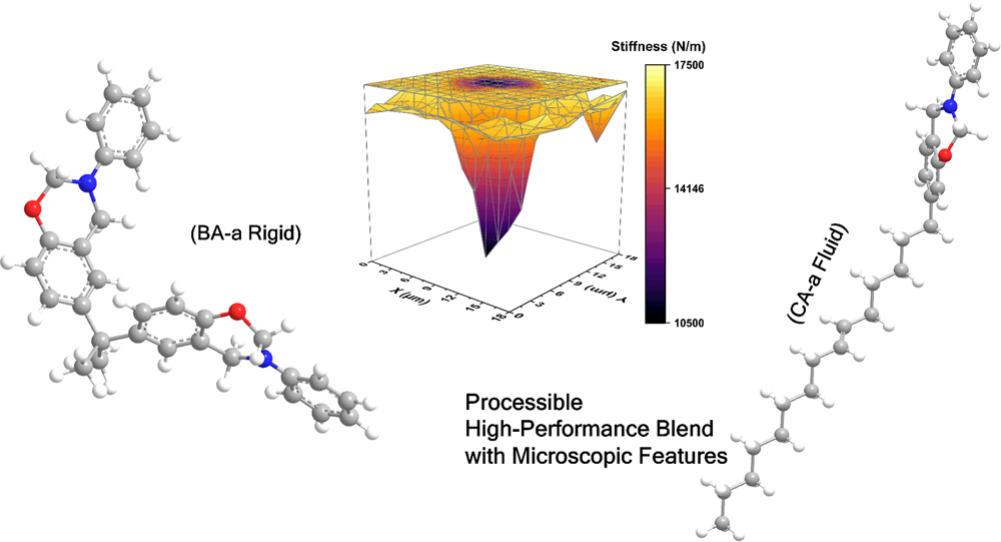

A benzoxazine system is presented combining liquid cardanol-based benzoxazine (CA-a) and an effective initiator (3,3′-thiodipropionic acid, TDA) to bisphenol A-based benzoxazine (BA-a). The resultant mixture of monomeric precursors shows excellent fluidity and a relatively low peak polymerization temperature of around 200 °C. Moreover, the cured polybenzoxazine displays a high thermal decomposition temperature (*T*_d,5%_ > 330 °C), a moderately high glass transition temperature (∼148 °C), and robust mechanical strength (storage modulus ∼ 2.8 GPa) comparable to those of the polybenzoxazine homopolymer obtained by curing BA-a. A comprehensive investigation into the microstructure and curing kinetics has also been conducted on the system, offering an extensive background for future studies.

## Introduction

Polybenzoxazine (PBz) resins possess advantageous properties over traditional thermoset polymers such as epoxy or phenolic resins, e.g., excellent polymerization dimensional stability, low water absorption, good thermal mechanical properties, and high-temperature resistance.^1−4^ As a result, PBz resins, with properties first reported in 1994,^5^ have attracted more and more attention from both a scientific and a technical perspective. Such promising materials can be strong contenders to complement or even supplant existing polymers such as polyesters, epoxies, phenolics, and bismaleimides, with the potential to be used in the aerospace, transportation, electronics, as well as the oil or gas industries.^6^ However, unlike other conventional resins, benzoxazines are not yet widespread in the industry, and there is a lack of material databases and common building blocks. Moreover, the complicated processing caused by the solid nature of the monomer and its poor reactivity are still problems limiting its wider usage.^6^ Therefore, there is a need for systematic development of well-understood and facile processing of benzoxazine systems to further develop this class of high-performance resins.

To improve the processability, researchers investigated different materials as diluents, such as low-viscosity benzoxazine resins,^7−9^ epoxy resins,^7,10^ or other reactive diluents.^9^ To lower the elevated curing temperatures (>200 °C) and shorten the polymerization time, several different approaches have been proposed, including incorporation of initiators,^11−13^ design of self-catalytic monomers,^14−16^ and utilization of intermolecular association.^17^ There have been various studies focusing on a specific problem; however, few comprehensive studies have been conducted on a benzoxazine system that is liquid-processable with reduced curing temperature, without compromising its thermal and mechanical performance.

Bisphenol A-based benzoxazine (BA-a) is one of the earliest and most widely reported monomeric benzoxazines achieving cross-linked polymers,^5,18^ showing glass transition temperatures around 160 °C and Young’s moduli over 3 GPa.^12,19^ However, this benzoxazine monomer is solid at room temperature, which makes it difficult to introduce and fully disperse any micro- or nanoenhancers, as well as to employ automatic composite fabrication methods such as resin transfer molding (RTM) and filament winding. The monofunctional benzoxazine (CA-a) is a bioderived-benzoxazine obtained from cardanol, which is a byproduct of the widely used natural material cashew nutshell liquid (CNSL);^20,21^ the high fluidity of this prepolymer is advantageous for processing. However, its chemical structure prevents it from undergoing extensive network formation, which makes the largely linear poly(CA-a) show less favorable mechanical and thermal properties compared to other conventional difunctional benzoxazines. 3,3-Thiodipropionic acid (TDA) has been found to be an excellent initiator, offering a rapid polymerization mechanism and a higher glass transition temperature in the BA-a benzoxazine system.^12^

In this work, different blends of BA-a, CA-a, and TDA were prepared and investigated to develop a benzoxazine system with good handling capability without significant sacrifice of the thermal and mechanical properties.

## Materials and Methods

### Materials

Benzoxazine Araldite MT35600 (hereafter BA-a) and benzoxazine Araldite MT35500 (hereafter CA-a) were kindly provided by Huntsman Advanced Materials (Basel, Switzerland), and TDA >97% was purchased from Merck.

All materials were used as received without any purification.

### Preparation of a Modified Benzoxazine System

For BA-a/CA-a samples, solid BA-a was ground into a fine powder using a mortar and pestle and then added to liquid CA-a. The resin mixtures were then mixed manually in a metal beaker using a spatula. Afterward, the resin blends were stirred using an IKA-WERKE EUROSTAR digital overhead stirrer with a Cowles blade at 1000 rpm. During the whole process, the temperature was kept at 100 °C using an IKA RCT classic hot plate equipped with an oil bath. The mixture was stirred for 15 min, at which point it was homogeneous. Five formulations were explored for the BA-a/CA-a system.

The initiator (TDA) was incorporated into samples after the resin had been blended. Another 15 min mixing period was added to allow the even dispersion of the initiator. Four formulations (Table S1) were explored for the Bz/I system, and usually, approximately 60 g samples were made each time.

### Rheology

Rheological measurements were carried out using a TA Discovery HR 10 rheometer. For all samples, dynamic scans were performed at a heating rate of 2 °C min^–1^ from 40 to 180 °C using 25 mm ETC aluminum disposable parallel plates. A higher starting temperature of 60 °C was utilized for the solid resin BA-a.

### Differential Scanning Calorimetry (DSC)

DSC experiments were performed using a Netzsch 204 F1 DSC. Sealed aluminum pans were used, with sample masses of around 5 mg.

For basic polymerization behavior research, samples were heated from 30 to 300 °C at a heating rate of 6 °C min^–1^.

For specific kinetics studies, samples were treated between 30 and 300 °C at heating rates of 3, 6, 10, 15, and 20 °C min^–1^. The temperatures of the polymerization exotherm peak were recorded, and Kissinger, Ozawa, and Friedman methods^22,23^ were used to determine the activation energy and other kinetic parameters.

Sample cells were kept under a constant flow of dry nitrogen (50 mL min^–1^) for all the testing.

### Thermogravimetric Analysis (TGA)

The simultaneous thermal analyzer NETZSCH STA 449 F3 Jupiter was used on the specimens to determine the thermal stability and char yield differences. Ceramic pans were used with sample masses of around 10 mg. All the specimens were scanned from 30 to 1000 °C under a flow of nitrogen at a heating rate of 10 °C min^–1^.

Samples used for TGA were cured at 180 °C for 2 h and 200 °C for 1 h with a ramping rate of 2 °C min^–1^. A post cure at 220 °C for 1 h was added for CA-a to ensure a high degree of conversion.

All samples were tested in triplicate, and an average was calculated.

### Dynamic Mechanical Thermal Analysis (DMTA)

The DMTA tests were performed with a DMA Q800 from TA Instruments with a heating rate of 5 °C min^–1^ from room temperature to 250 °C. The analysis was carried out in a single cantilever mode at a frequency of 1 Hz and a strain with an amplitude of 15 μm.

The samples were directly cured to a rectangular shape in a silicone mold with dimensions approximately 39.5 mm × 14.5 mm × 2.5 mm. Samples used for DMTA were cured at 180 °C for 2 h and 200 °C for 1 h with a ramping rate 2 °C min^–1^. A post cure at 220 °C for 1 h was added for CA-a to ensure a high conversion.

### Microindentation

The FT-MTA03 micromechanical testing and assembly system from FemtoTools AG was utilized on samples for microindentation characterization, equipped with an FT-S2000 microforce sensing probe. A spherical tip was obtained by attaching a borosilicate glass microsphere, with a radius of 22 μm, to the silicon probe. The samples were indented using the piezoscanner at a speed of 1 μm s^–1^, and indentation was carried out until a maximum force of ∼ 2000 μN was reached.

The loading and unloading processes were both recorded, and the integrated instrument software was used to analyze the data. The loading curve was used to calculate the stiffness for the region of the curve between 10 and 90% of the maximum force.

## Theoretical Basis of Curing Kinetics

Generally, DSC is a common method to examine the curing kinetics of thermoset polymerization reactions, which assumes that the obtained heat flow  is proportional to the reaction rate. Based on this assumption, eq 1 can be obtained:^24^
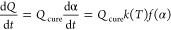
1where *t* is the time (s), *Q*_cure_ is the total exothermic heat for the sample being fully cured (J), α is the degree of cure, indicating the extent of a monomer conversion to the polymer, *k*(*T*) is the temperature-dependent reaction rate constant, while *f*(α) is the differential conversion function depending on the polymerization mechanism.

Owing to the autocatalytic character of the benzoxazine system,^25^*f*(α) can be described as follows (eq 2), attributed to the Šesták–Berggren reaction model [SB(*m*,*n*)]:^26^
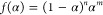
2where *n* and *m* are reaction orders.

For *k*(*T*), the Arrhenius equation can be used:^27^
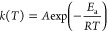
3where *A* is the preexponential factor (s^–1^), *E*_a_ is the activation energy (J mol^–1^), *T* is the absolute temperature (K), while *R* is the universal gas constant (J K^–1^ mol^–1^).

As the dynamic scanning is performed here, the temperature *T* can be calculated based on the time *t* (s) and ramping rate β (K s^–1^); the following equations are obtained:
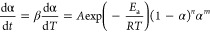
4
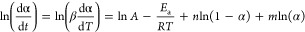
5

All of the methods used for determining kinetic parameters in this study will be based on those equations.

The Kissinger method^28^ and Ozawa method^29^ are very popular methods of calculating the activation energy of thermally stimulated processes. In this study, they were utilized to determine how the catalyst changes the average activation energy of the whole curing process without detailed knowledge of the kinetics.

The Kissinger method assumes the maximum reaction rate corresponding to the polymerization peak point. As the derivative of reaction rate versus time for that point is 0, the following expressions (eqs 6 and 7) can be used:
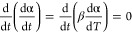
6
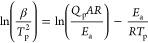
7where .

Therefore, the activation energy can be determined from the plot of  versus .

The Ozawa method, modified employing the Doyle’s approximation of the temperature integral,^30^ is similar to the Kissinger method, but based on the linear relationship between ln β and  (eq 8):
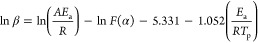
8where .

It should be noted that both Kissinger and Ozawa methods can only give an average *E*_a_ value representing the general level of the reaction. However, the *E*_a_ value differs with the degree of cure. Therefore, the Friedman^31,32^ method is then used to calculate the apparent activation energy E_α_, which is the activation energy for different stages during the reaction. The Friedman method is based on eqs 4 and 5. When the conversion α is a certain value, *E*_α_ can be determined from the plot of  versus .

## Results

### Formulation Determination and Basic Properties of Modified Benzoxazine Systems

To form a benzoxazine system with enhanced processability, two vital factors should be considered: the liquefaction point and the gel point. Generally, two main events can be clearly seen in a typical evolution of the viscosity of resin versus temperature (Figure S1). The liquefaction point occurs in a relatively low-temperature range, which is related to the transition of resin from solid to liquid, while the transition that happens at higher temperature is the gel point, representing the gelation and polymerization of the monomeric compounds. The range between these two points is the suitable manufacture temperature range, which is also called processing window.^33^ For comparison, the temperature point at which the viscosity value reaches 500 Pa s was used to determine the liquefaction point and the gel point.

The determination of the sample formulation could be divided into two steps based on the theory mentioned above: first, to lower the liquefying temperature and expand the processing window, a suitable type and amount of liquid monomer was incorporated into a solid benzoxazine system. After that, considering the manufacturing difficulty caused by high polymerization temperature and gel point, an efficient initiator was selected to increase the reactivity of the benzoxazine system. Rheology, DSC, TGA, and DMTA have been carried out to understand the performance of various specimens, making the whole system liquid-processable without sacrificing its other properties.

**Figure 1:**
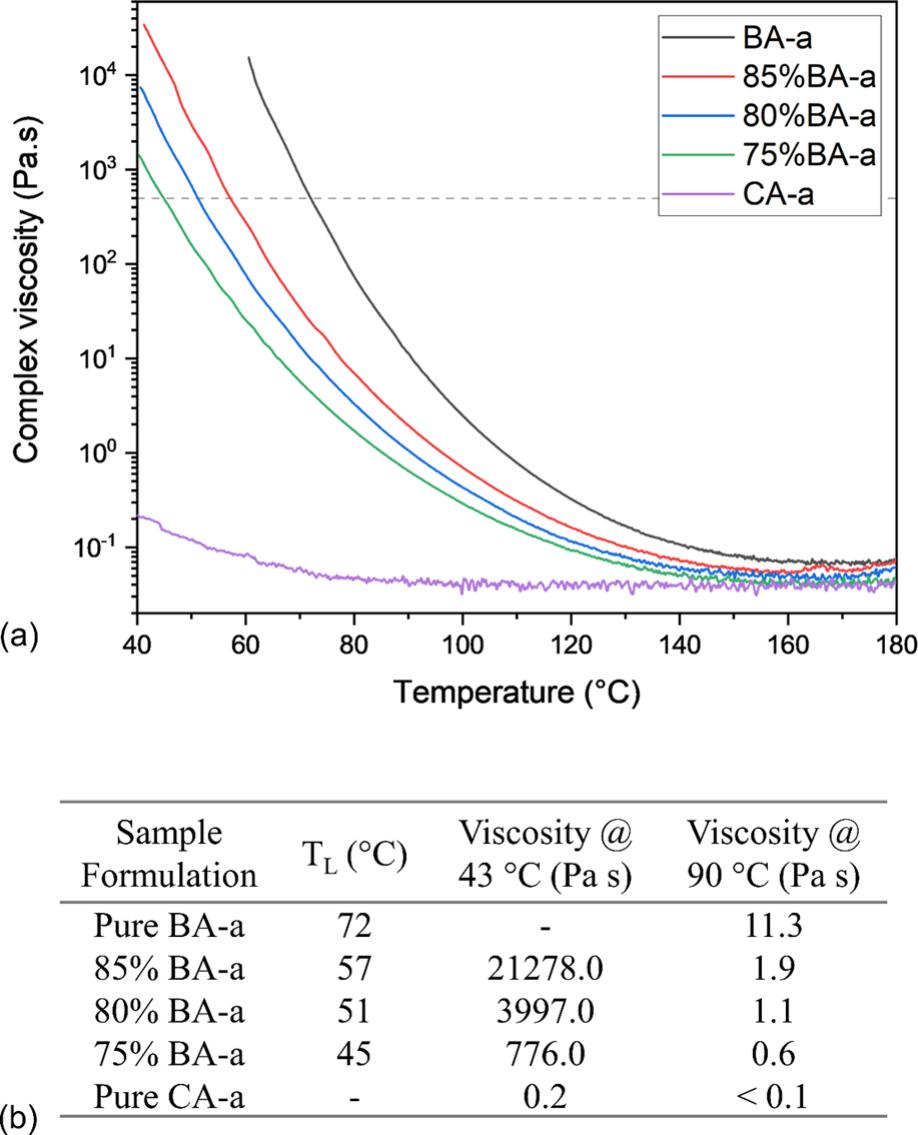
Rheological results of BA-a/CA-a systems and (a) dynamic rheological scanning curves. The dashed line indicates a viscosity of 500 Pa s, the threshold below which samples are considered liquid: (b) detailed liquefaction temperature and specific viscosity values of samples at different temperatures.

For the first step, the fluidity of the system was considered. Dynamic rheological scanning was performed on samples with different fractions of CA-a. Figure 1a shows the rheological properties of the pure BA-a and CA-a samples as well as those of mixtures with different formulations. In the considered window, the viscosity of CA-a is low (i.e., < 0.3 Pa s) and remains unchanged from 80 °C (<0.1 Pa s), while all the other samples show an obvious progressive reduction in viscosity with increasing temperature. Temperature at the viscosity value of 500 Pa s was used as the liquefaction temperature (*T*_L_) for comparing different resin systems, which are shown in Figure 1b. Overall, the addition of CA-a significantly increases the fluidity of the benzoxazine system and makes the whole system liquid-processable even at a relatively low temperature of around 45 °C.

The liquefaction temperature and viscosity values match well with those in the literature. For example, Rimdusit et al.^33^ found that the *T*_L_ for BA-a is 76 °C, which is close to 72 °C obtained here. A dynamic viscosity of BA-a was determined as 26 Pa s at 90 °C, which is in the same magnitude as ∼ 11 Pa s recorded in our study. Moreover, Lochab et al.^34^ achieved a viscosity value of 0.15 Pa s for pure CA-a at 43 °C, which is consistent with 0.2 Pa s obtained in our study. Slight differences can be attributed to the variances in the purity or testing parameters.

**Figure 2:**
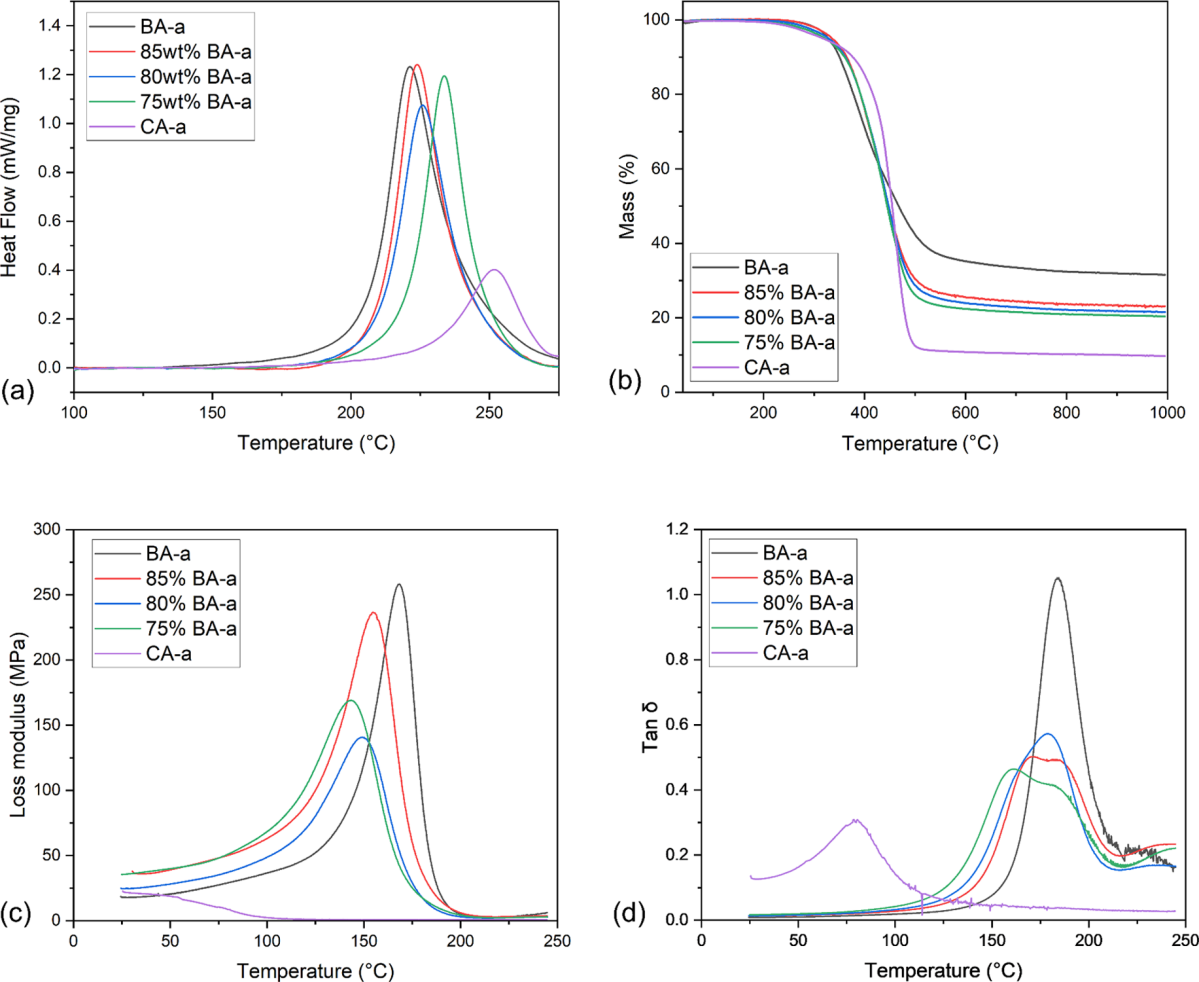
(a) DSC data for uncured Bz showing heat flow (exotherm upward) versus temperature, baseline subtracted using Origin for easier comparison; (b) dynamic TGA data for cured PBz showing residual mass versus temperature; (c) dynamic DMTA data for cured PBz specimens showing loss modulus versus temperature; (d) DMTA data for cured PBz specimens showing tan δ versus temperature.

The polymerization process of BA-a and CA-a, respectively, is shown as Scheme S1. The polymerization behavior, glass transition temperature, and thermal stability of the BA-a/CA-a systems were also briefly explored, and results are listed in Table 1 and Figure 2.

**Table 1:** Thermal Properties of the BA-a/CA-a System^a^

sample formulation	*T*_p_ (°C)	polymerization enthalpy		final residue (wt %)	*T*_g1_ (°C)	*T*_g2_ (°C)	tan δ peak height
		(J g^–1^)	(kJ mol^–1^ Bz ring)				
pure BA-a	221	361	84	32	168	184	1.05
85% BA-a	224	307	76	23	155	179	0.57
80% BA-a	226	273	69	22	149	168	0.51
75% BA-a	234	266	69	20	143	161	0.46
pure CA-a	252	91	38	10	∼ 30	80	0.31

a *T*_p_ = peak polymerization temperature; *T*_g1_ = glass transition temperature (loss modulus peak); *T*_g2_ = glass transition temperature (highest tan δ peak); tan δ peak height was determined from the highest peak; the final residue was recorded at 1000 °C.

Owing to the differences in the molecular structure between BA-a (Scheme S1a) and CA-a (Scheme S1b), the former shows a much higher polymerization enthalpy compared to the latter (Table 1). The polymerization enthalpies found here are comparable to the literature values, which show Δ*H* values of 360.0 and 74.0 J g^–1^ for BA-a and CA-a, respectively.^8^ The differences between the CA-a enthalpies might be related to the use of different side chains and to the purity of reagents. In addition, it is somehow surprising to see that the Δ*H* (J mol^–1^ of Bz ring) between BA-a and CA-a is so different (BA-a approximately twice the value of CA-a), which might be attributed to the effect of steric hindrance afforded by the long flexible side chain in CA-a. Moreover, pure CA-a shows a significantly higher onset polymerization temperature and lower reaction rate at the beginning stage compared to pure BA-a (Figure 2a). While the former could be attributed to the aforementioned steric hindrance, the latter is due to the monofunctional features of the CA-a benzoxazine. In contrast, the quicker induced curing of BA-a can be explained by the higher concentration of phenolic groups at the ring-opening stage. For all the BA-a/CA-a mixtures, specimens show single exothermic peaks, indicating the copolymerization mechanism between the BA-a and CA-a benzoxazines. Meanwhile, with increasing loading of CA-a, the blends show a high-temperature-shifted polymerization peak, as well as a decreased enthalpy, which may be attributed to the steric effect caused by the alkenyl chain of CA-a.^8^

From the TGA curves (Figure 2b), a consistent three-stage weight loss process can be found for all specimens: From room temperature to about 300 °C, the mass of samples only shows a minor reduction; as the temperature rises further, samples lose mass rapidly; when the temperature goes over 600 °C, the mass of the samples stabilizes. For BA-a, the initial degradation phase could be attributed to amine evaporation, while the major degradation stage is related to the simultaneous degradation of the phenolic linkage and the Mannich base. CA-a shows a higher degradation temperature, which could be attributed to the smaller proportion of the Mannich bridge in the compound.^35,36^ Moreover, CA-a shows a much quicker degradation after 450 °C compared to BA-a, which is due to weaker methylene linkages in the alkyl side chain of the cardanol moiety.^8,34^ These variances between the two benzoxazines make the degradation process of these copolymerized samples appear very similar at first glance; however, some non-negligible differences can be observed: Generally, a higher concentration of CA-a tends to leave samples with less residue at 1000 °C while enhancing the thermal stability of the samples in the range of 350–450 °C.

The glass transition temperature (*T*_g_) values were obtained from the loss modulus versus temperature, as well as the tan δ versus temperature curves (Figure 2c,d), for a wider comparison. The BA-a and copolymerized samples show *T*_g_ values ranging from 143 to 168 °C, which is comparable, if only slightly higher, than the values obtained from previous studies.^8,12^ The *T*_g_ of CA-a extracted from the loss factor (tan δ) curve is 80 °C, which is also consistent with prior publication values, i.e., 89 and 77 °C.^37,38^ The copolymerized 75% BA-a sample shows a *T*_g_ of 143 °C, which is 25 °C lower than that of the pure BA-a homopolymer. Moreover, the addition of CA-a broadens the loss modulus peak while lowering its intensity. The former can be explained by the copolymerization of CA-a monomers with BA-a, generating more variable structures compared to the pure BA-a system, while the latter can be attributed to the hindrance and entanglements effects induced by the cardanol long chain. Finally, the shape of the tan δ peaks changes significantly (Figure 2d). The increasing loading of CA-a leads to broader peaks compared to that of the pure BA-a system, indicating more complicated molecular microstructure distributions inside the copolymerized samples and greater damping. In particular, the 75% BA-a sample shows two tan δ peaks centered around 160 and 180 °C. This phenomenon may reflect the presence of two slightly different phases in the sample, which are a CA-a/BA-a copolymer and BA-a-dominated (nearly single-component) phase, respectively. It is common to see such trends with tan δ curves, for example, Rao and Palanisamy^39^ prepared a CA-a/cardanol epoxy copolymer and observed a broader tan δ peak compared to the homopolymerized samples. The amplitude of damping is related to the energy dissipation capacity of the material, of which values are indicated in Table 1. The addition of CA-a induces completely opposite effects in the low- and high-temperature ranges: Samples with CA-a show higher damping in the glassy state but lower damping in the viscoelastic state. This can be explained by the long side chain in CA-a, which is frozen at room temperature but begins to move freely and disturb the movement of neighboring molecules with the increase in temperature.^8^ The values of the tan δ peak height of CA-a and BA-a are 0.31 and 1.05, respectively, which is comparable to the literature results 0.30 and 0.90.^12,39^

The 75% BA-a sample, offering the best processability (i.e., lowest viscosity) and comparable thermal properties as BA-a, was thus selected for further studies and hereafter named pure Bz for step 2. During this step, samples with various fractions of the initiator, TDA, were prepared (from 0 to 3 wt %). The goal of this stage is to lower the curing temperature of the system and compensate the properties sacrificed by the addition of the CA-a.

The basic polymerization behavior of monomeric materials and thermal stability of the cured resins were first investigated, and the results are listed in Table 2.

**Table 2:** Thermal Properties of the Bz/I System^a^

sample formulation	*T*_p_ (°C)	polymerization enthalpy		final residue (wt %)	*T*_g1_ (°C)	*T*_g2_ (°C)	tan δ peak height
		(J g^–1^)	(kJ mol^–1^ Bz ring)				
pure Bz	234	266	69	20	143	161	0.46
Bz 1I	211	291	77	20	145	163	0.37
Bz 2I	201	297	79	19	148	186	0.38
Bz 3I	196	258	69	20	145	181	0.44

a *T*_p_ = peak polymerization temperature; *T*_g1_ = glass transition temperature (loss modulus peak); *T*_g2_ = glass transition temperature (highest tan δ peak); tan δ peak height was determined from the highest peak; the final residue was recorded at 1000 °C.

**Figure 3:**
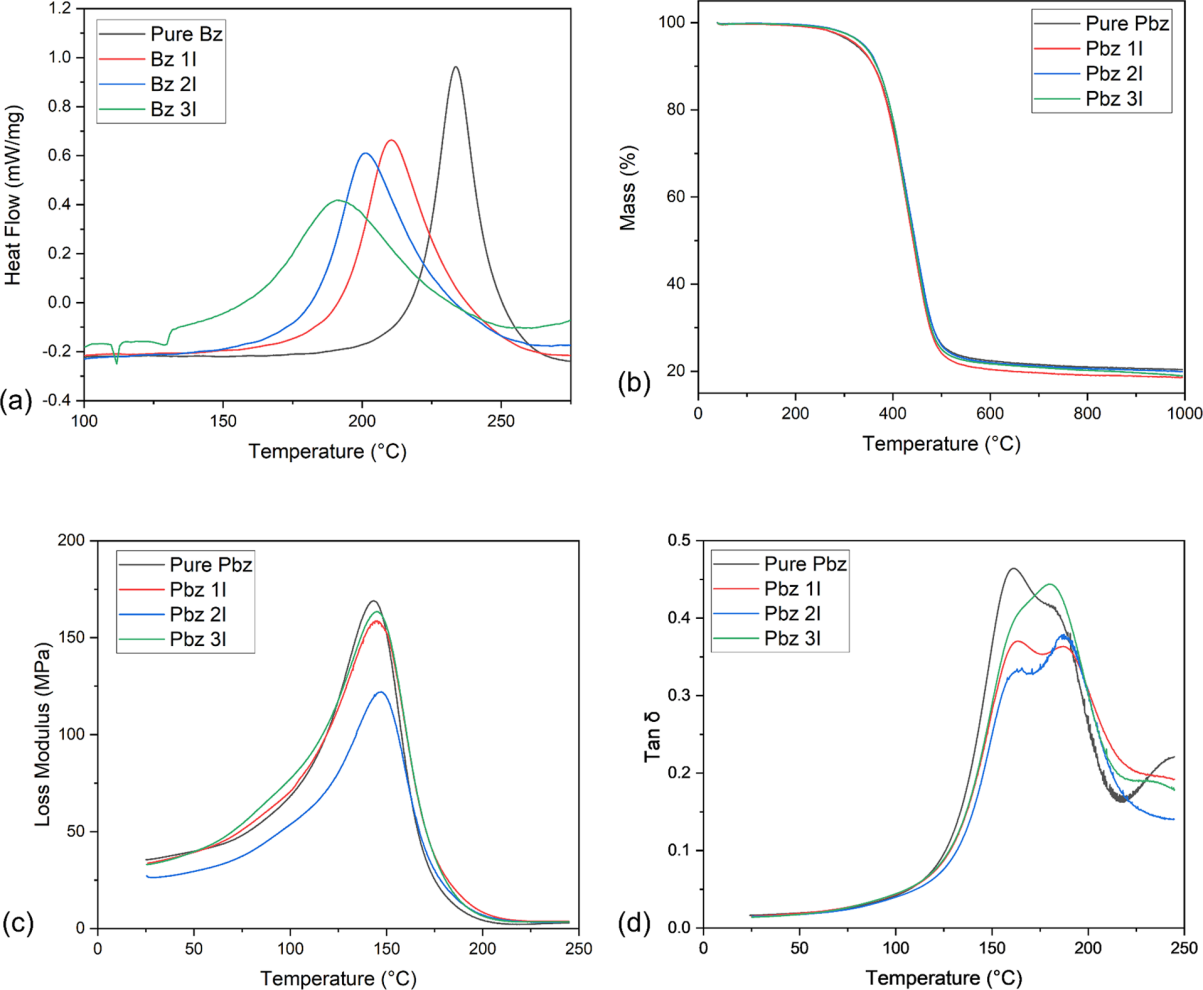
Thermal/mechanical properties of the Bz I system; (a) DSC curves, baseline subtracted using Origin for easier comparison; (b) TGA curves; (c) temperature–loss modulus curves obtained from DMTA; (d) temperature–tan δ curves obtained from DMTA.

From the DSC experiments (Figure 3a), it could be seen that the polymerization peaks are broadened, become more asymmetrical, and are shifted toward lower temperatures with the increase of the TDA loading. These phenomena are observed because TDA plays a more important role at the initial ring-opening stage rather than in the later steps.^12^ Moreover, the Bz 2I samples show 14% higher polymerization enthalpies compared to the uninitiated samples, indicating that more highly cross-linked networks might be formed. Compared to previous work,^12^ in which the 2 wt % initiator only increases the polymerization enthalpy by 8%, it is apparent that TDA is more effective for samples in this study. This phenomenon is expected as CA-a is more sensitive to the incorporation of TDA compared to BA-a (Figures S2 and S3). It should be noted that the polymerization enthalpy of Bz 3I listed here is likely underestimated compared to the actual value; there is a very significant melting peak of the TDA around 130 °C, which makes the determination of the onset polymerization temperature and complete polymerization enthalpy difficult.

From the TGA curves (Figure 3b), the samples with an initiator tend to degrade at higher temperatures, especially PBz 2I and PBz 3I, which lose 5% of their mass at a temperature over 15 °C higher than that of pure PBz. However, there are no obvious changes between the char yields among various samples. The former phenomenon could be related to the formation of stronger networks with more cross-linkages in the PBz I samples, while the latter may be attributed to the degradation of TDA itself. Generally, there are no significant changes in the thermal stability of the samples with the addition of TDA.

The glass transition temperatures of the specimens are obtained from the plots of loss modulus versus temperature curves obtained from DMTA data (Figure 3c); all the initiated samples show increased *T*_g_ values, especially, the PBz 2I sample owns the highest *T*_g_ at 148 °C. Moreover, the shapes of the tan δ peaks are also changed with the incorporation of TDA (Figure 3d). For the uninitiated sample, it shows a higher CA-a/BA-a copolymer tan δ peak centered at 150 °C, accompanied by a slightly lower BA-a-dominated peak around 180 °C. However, the CA-a/BA-a copolymer peaks become relatively less intense compared to the BA-a-dominated peaks with the addition of a more catalyst. This might be because TDA leads to more ring opening of CA-a compared to that of BA-a, which allows CA-a to react preferentially with more BA-a to form a more rigid copolymer, which lowers the intensity of the tan δ peak.

It is not uncommon to see that the area of tan δ peaks of different microphases changes with different curing processes. For example, Abdouss et al.^40^ reported the tan δ peak changes of the epoxy-polysulfide copolymer following longer curing times.

The formulation of Bz 2I was selected for use in further studies as it shows a low liquefaction temperature (∼47 °C, Figure S4), moderate polymerization temperature (*T*_p_ = 201 °C), higher glass transition temperature (148 °C), robust mechanical strength (storage modulus ∼ 2.8 GPa, Figure S5), as well as a thermal stability comparable to that of the starting benzoxazine (BA-a).

### Detailed Polymerization Process Analysis

**Figure 4:**
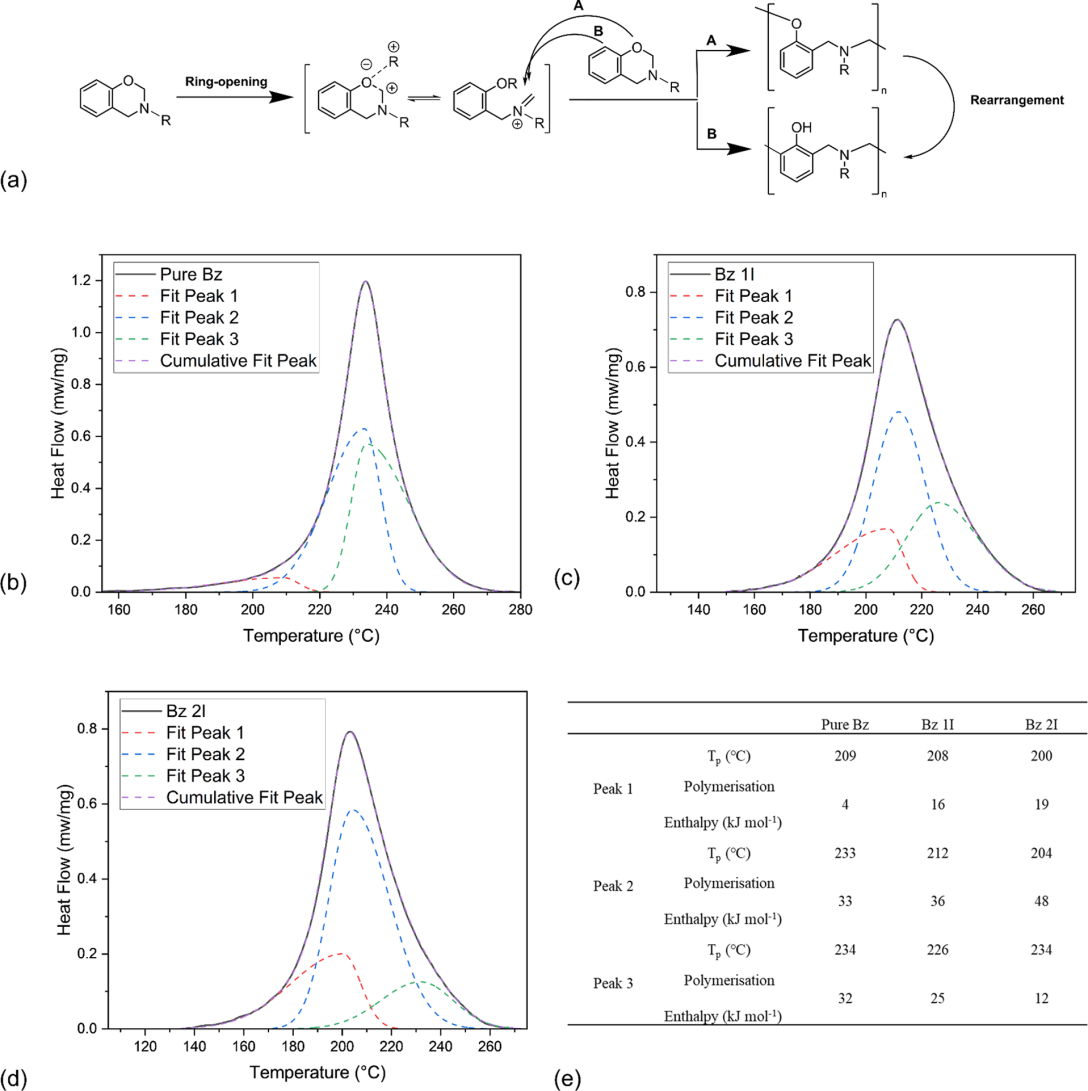
(a) Schematic polymerization process of benzoxazine. Adapted with permission from ref (43) (American Chemical Society, 2011); peak fitting for the specific samples (b) pure Bz, (c) Bz 1I, and (d) Bz 2I; (e) peak parameters of each stage of polymerization, calculated from the deconvoluted peaks shown in (b)–(d) graphs.

The polymerization peaks of pure Bz, Bz 1I, and Bz 2I acquired from DSC analysis were deconvoluted and fitted with FityK using a Split Gaussian function to see how the initiator affects each stage of the curing process.^41^ Overall, all the samples show clear three-stage patterns (Figure 4a), with peaks that can be assigned to the ring opening, bridge forming, and structural rearrangement from low to high temperature, respectively.^12,42^

As shown in Figure 4, peak 1 is related to the ring opening. It becomes larger and peaks at lower temperatures with increasing initiator loading, indicating the great influence of TDA in the early stages of polymerization. Moreover, the peaks related to the following polymerization phases tended to also start at lower temperature when the ring-opening temperature decreased. Meanwhile, not only has the onset temperature of each stage been affected, but the enthalpy of each phase has also been changed. As shown in Figure 4e, the peak related to the ring opening changes the most, and the corresponding enthalpy rapidly increases by more than 3 times with only 2 wt % TDA. This phenomenon offers strong evidence that such acid initiators bring the largest influence on the cleavage of the ring in the polymerization reaction. In contrast, the structural rearrangement was deactivated by the presence of an initiator, which might be due to the fact that rapid ring opening led to a dramatic increase in the viscosity of the samples, making the movement and rearrangement of the molecule more difficult. Meanwhile, the enthalpy of the bridge forming showed some improvement in the presence of TDA. Also, the peak temperature of the three stages becomes closer with higher loading of the catalyst, indicating that these processes tend to happen simultaneously during the polymerization.

Vibrational spectroscopy has been used to monitor the polymerization behavior of both pure BA-a and BA-a/5 mol % TDA systems in a previous study,^12^ where changes in the characteristic peaks match quite well with our observations.

### Curing Kinetics Study

Isothermal DSC testing (Table S2, Figure S6) has been carried out on Bz 2I samples to determine a suitable curing schedule: 180 °C for 2 h and 200 °C for 1 h with ramping rates of 2 °C min-1, after which curing was found to be complete.

**Figure 5:**
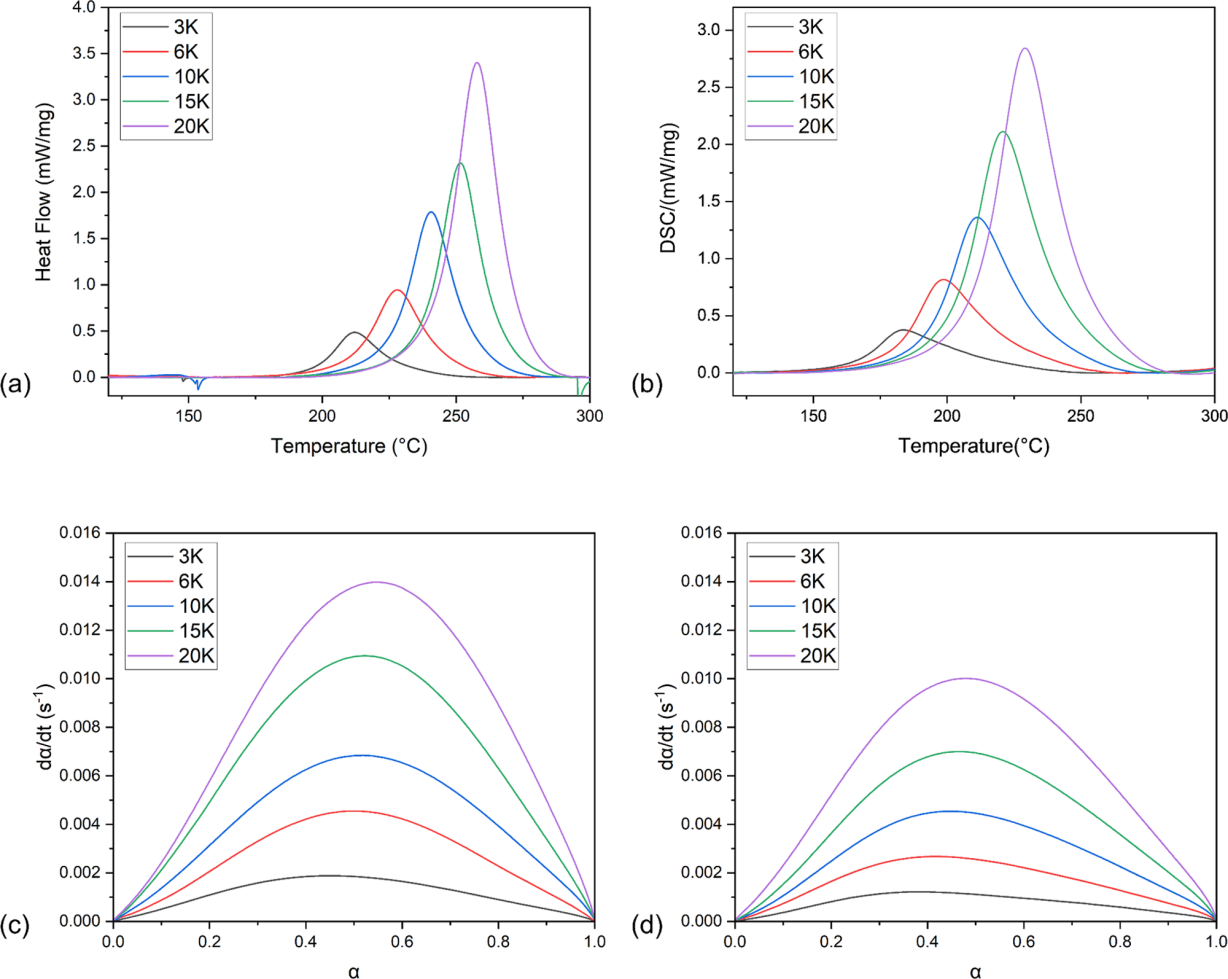
Plots of DSC curves showing reaction rate versus conversion with different ramping rates of (a) pure Bz, (b) Bz 2I; reaction rate versus degree of cure of (c) pure Bz and (d) Bz 2I.

A further curing kinetics study was performed with the samples of uninitiated pure Bz and Bz 2I using multiple DSC scans with different heating rates (Figure 5a,b). The curves showing the reaction rate as a function of the conversion were also obtained (Figure 5c,d), in which the maximum reaction rate for the system is in the range of 20–60% conversion, indicating that the sample follows an autocatalytic mechanism, rather than *n*th-order kinetics.^25^ Such a finding is not unexpected as the benzoxazine can generate a large concentration of highly catalytic – OH groups with the increase in the degree of cure.^44^

**Figure 6:**
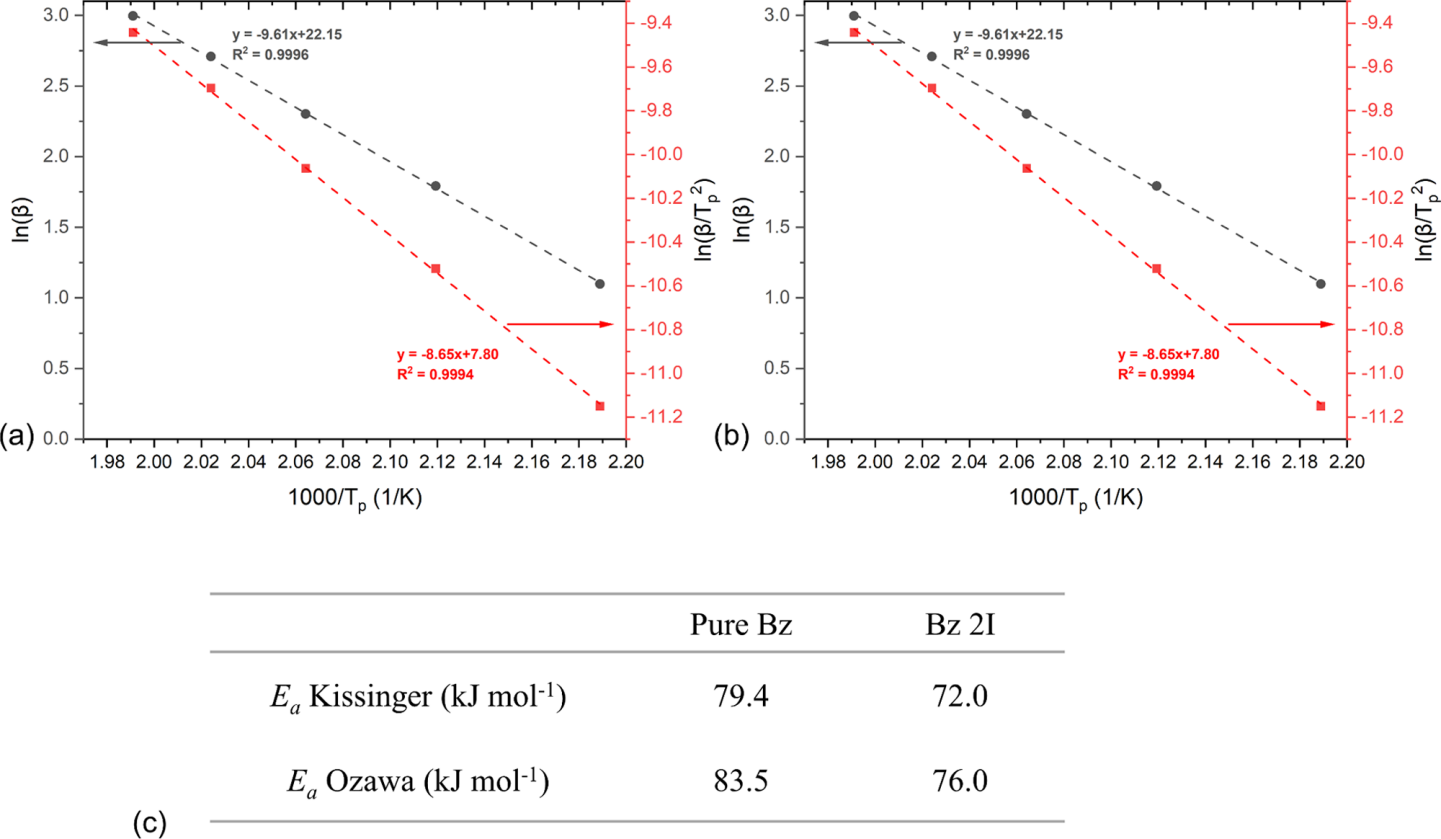
Kissinger plot (red square) and Ozawa plot (black circle) of (a) pure Bz; (b) Bz 2I; (c) calculated activation energy of samples.

To calculate the activation energy of the samples easily, Kissinger and Ozawa methods were used; the plots and results of these two methods are shown in Figure 6. The *E*_a_ values were in reasonable agreement with those found in the literature, in which the activation energies for the two components BA-a and CA-a were determined as 81.4 and 118 kJ mol^–1^, respectively.^12,37^ Our slightly lower activation energies could be attributed to the oligomers generated during the high-temperature mixing in which the phenolic groups increase the reactivity. Moreover, it is clearly seen that the activation energy of the polymerization reaction drops with the addition of the TDA initiator, which makes the concentration of the activated monomer in the Bz I sample become higher compared to the sample without a catalyst at the same temperature.

Based on the *E*_a_ obtained from the Kissinger method, the detailed kinetic parameters could then be determined using multiple linear regression using eqs 4 and 5. As the kinetic parameters change significantly with the increase of the degree of cure and the associated rise in viscosity, it is important to select a suitable range to do the fitting. The parameters were finally calculated at 20–40% conversion to take the autocatalytic nature of the system into account (Table 3).

**Table 3:** Detailed Kinetic Parameters Calculated from Each Scanning at 20–40% Conversion

heating rate (°C min^–1^)	pure Bz				Bz 2I			
	*n*	*m*	*n* + *m*	ln *A*	*n*	*m*	*n* + *m*	ln *A*
3	1.98	1.19	3.17	15.53	3.12	1.32	4.44	15.00
6	1.39	1.02	2.41	15.15	2.56	1.25	3.81	14.89
10	1.29	1.16	2.45	15.36	2.19	1.25	3.44	14.78
15	1.43	1.27	2.70	15.57	2.00	1.24	3.24	14.77
20	1.08	1.16	2.21	15.31	1.73	1.15	2.88	14.62
average	1.43	1.16	2.59	15.40	2.32	1.24	3.45	14.82

The kinetic equation of these two systems (20–40% conversion) can then be expressed as
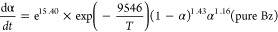

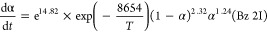


Generally, Bz 2I shows a higher total index (*n* + *m*), indicating a quicker polymerization process. In details, the *n* exponent representing the reaction rate is proportional to the unreacted material (1 – α), indicating the chemically controlled part of the reaction, while the *m* parameter is related to the reacted material α, reflecting the autocatalytic effect and diffusion controlled part.^31,44^ Bz 2I shows a higher value of n compared to that of pure Bz, which is also consistent with the fact that TDA accelerates the ring cleavage of the uncured benzoxazine. The two systems show similar *m* values, though the specific value of pure Bz system is slightly lower, which might come from the fact that pure Bz tends to form longer chains, due to the limited activated monomers at the early stage of the curing process, leading to a quicker viscosity increment.

These values are in reasonable agreement with those found in the studies by Hamerton et al.^12^ and Ambrožič et al.,^37^ which reported *n* values of 2.49 and 0.93 for BA-a and CA-a, respectively. It is not surprising for the pure Bz sample to show an intermediate *n* value as it is composed of BA-a and CA-a. Moreover, the ln *A* value of the pure Bz, which reflects the contact frequency of reactant molecules, is also found between the determined values of BA-a (9.31) and CA-a (23.03).^23,37^

Furthermore, the activation energies at various conversions were calculated based on the Friedman method^32^ to help explain the mechanistic differences between the two systems at different stages of the polymerization (Figure S7). The pure Bz sample generally shows a gradual increase in *E*_a_ with the increase in the degree of cure, which could be explained by the increasing viscosity of the samples during the curing process. The activation energy *E*_a_ drops slightly at around 25% conversion and the growth in the *E*_a_ slows thereafter, which can be attributed to the autocatalytic effects of the benzoxazine. In contrast to the pure Bz system, Bz 2I shows a slightly different trend: the curve shows a much smaller slope at the initial stage (α < 20%), which reflects the benefits conferred by the TDA initiator. However, the *E*_a_ of this system gradually exceeds that of pure Bz at higher conversion (α > 40%), which can be related to the higher cross-linked network formed inside the former (Bz 2I) system. These findings are also consistent with the literature, which also reveal that the activation energies of BA-a and CA-a will increase as the conversion increases.^32,45^

### Microstructure

**Figure 7:**
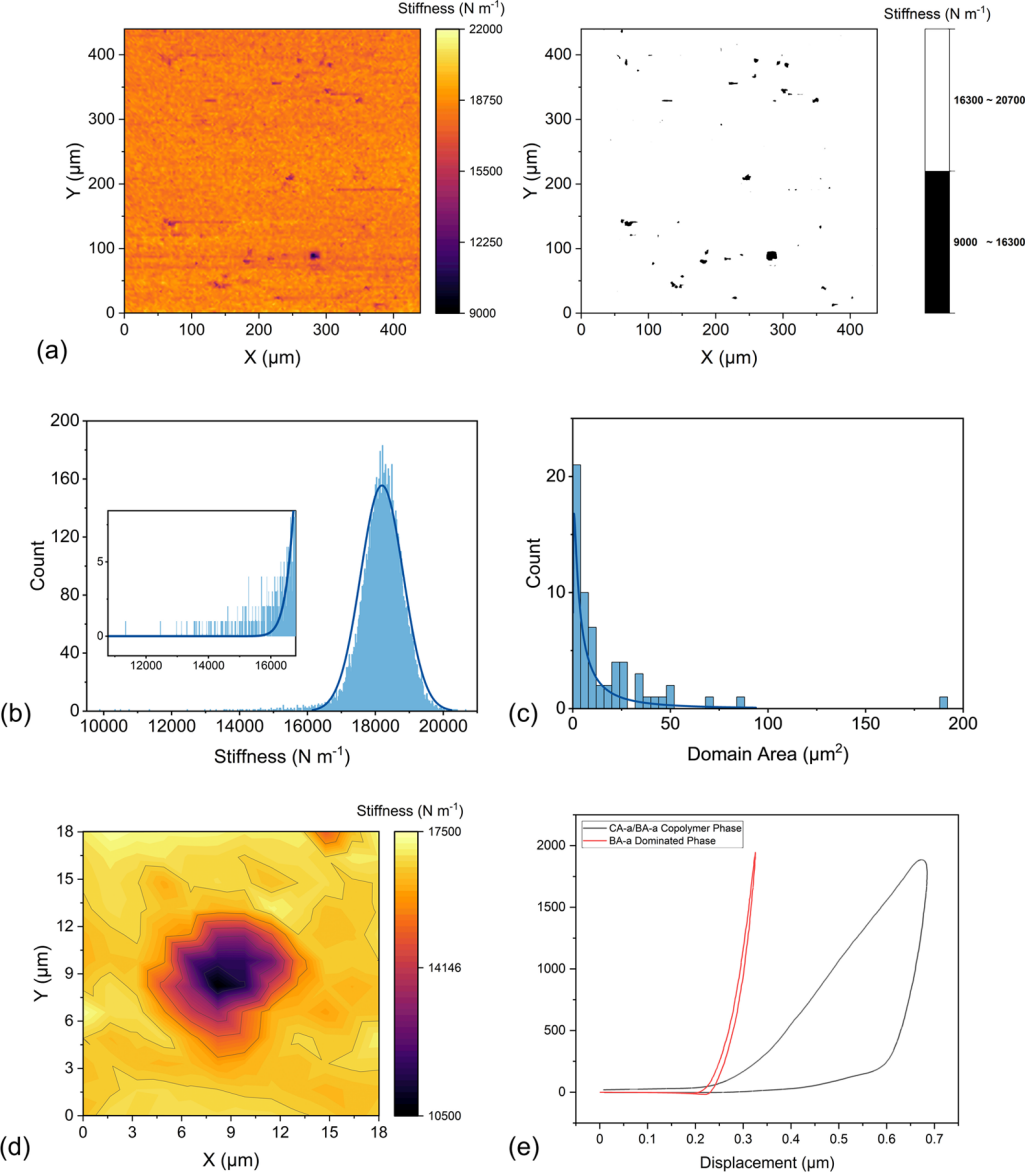
(a) Plots of stiffness data for a 440 μm × 440 μm domain in the Bz 2I sample; (b) histogram representing stiffness data for the Bz 2I sample; (c) histogram representing the domain sizes of the CA-a/BA-a copolymer area, log-normal curve used for distribution; (d) plots of stiffness data for an 18 μm × 18 μm area covering a single low stiffness domain in the Bz 2I sample; (e) plots of typical indentation curves (force versus displacement) of a CA-a/BA-a copolymer phase and BA-a-dominated phase.

Although no obvious structure differences are shown in the FTIR spectra (Figures S8 and S9), different microphases were indicated by the DMTA results. Therefore, microindentation experiments were performed on samples to extract more details about the different phases in the copolymerized BA-a/CA-a sample (Bz 2I). First, a large area (440 × 440 μm) was scanned to investigate both the relative occurrences and average areas of the CA-a/BA-a copolymer and BA-a-dominated domains. As shown in Figure 7, the bulk materials show a high stiffness ranging between 16,300 and 18500 N m^–1^, while some small discrete areas show significantly lower stiffnesses in the 9,000–16,300 N m^–1^ range. These discrete phases can be attributed to the CA-a/BA-a copolymer areas, for which the lowest stiffness is about half that of the more highly cross-linked BA-a- dominated phase. Generally, the stiffness data here are comparable although slightly higher than the values reported elsewhere for microindentation analysis of polybenzoxazines (e.g., ∼ 10,000 and ∼ 7,000 N m^–1^),^46^ which is probably caused by the different size of probes used, as well as the differences between the molecular weights and structures of the monomers.

ImageJ was used to estimate the total area and average size of these CA-a/BA-a copolymer phases.^47^ To create a binary image, these phases were defined as those with a stiffness more than 3 standard deviations below the mean value for the normally distributed bulk value (i.e., < 16,300 N m^–1^). As shown in Figure 7c, the majority of such domains are quite small with an average size of around 17 μm^2^. The combined area of these unique phases is 1081 μm^2^, which accounts for only 0.56% of the overall scanned area.

A higher-resolution microindentation experiment was then performed over a smaller area (18 μm × 18 μm) to facilitate a more detailed study of the CA-a/BA-a copolymer phase. As shown in Figure 7d, there is no obvious border between the CA-a/BA-a copolymer phase and BA-a-dominant phase; the stiffness changes gradually with a diffuse transition zone. Figure 7e shows typical indentation curves for different domains, which reflect greater variation in these microphases in addition to the stiffness. The unloading behaviors of these two domains are totally different. For the BA-a domain, the load is almost completely reversible, indicating that this is a predominantly elastic region.^48^ However, for the copolymer domain, there is significant hysteresis between loading and unloading curves, indicative of a viscoelastic response.^49,50^

## Conclusions

A comprehensive investigation has been performed to develop an easily processable, high-performance benzoxazine system. The addition of CA-a makes the monomeric benzoxazine blends show a widened processing window thanks to a lower liquefying temperature. The incorporation of TDA as the initiator not only enhances the reactivity of the monomeric precursors but also allows compensation of the slight decline in properties, e.g., mechanical strength and glass transition temperature, caused by the incorporation of linear structures made of poly(CA-a). Moreover, the detailed study of the microstructure and curing kinetics paves the way for further modifications and manufacture of this system.

In summary, this work presents an important step toward improving the practicality of benzoxazine-based high-performance materials by making them liquid-processable and by lowering their curing temperature. However, while the introduction of CA-a lowers the cross-link density and will serve to increase the resilience of the cured resin to a degree, the intrinsic brittleness remains a drawback for PBz resins. Taking advantage of the processability of this system, the successful dispersion of toughening agents should be much easier and lead to the preparation of composites possessing an enhanced toughness. Research along these lines is currently in progress in our laboratories.

## Supporting Information Available

Sample formulation, schematic of the liquefaction point and gel point of a thermoset resin, chemical structure and polymerization scheme of BA-a and CA-a benzoxazines, DSC results of BA-a 2I and CA-a 2I samples, DMTA results of BA-a 2I and CA-a 2I samples, rheology data of the Bz 2I sample, storage moduli of samples, isothermal DSC of the Bz 2I sample, apparent activation energy of pure Bz and Bz 2I samples, and FTIR spectra of samples (PDF)
