# Supplementary material for: Properties and Curing Kinetics of a Processable Binary Benzoxazine Blend
Source: ACS Appl Polym Mater. 2023 Nov 15;5(12):10404–15. doi: 10.1021/acsapm.3c02221 (PMC10714347; doi:10.1021/acsapm.3c02221)
Supplement: Supplementary file 1 — ap3c02221_si_001.pdf [file ap3c02221_si_001.pdf]

Supporting Information

The Properties and Curing Kinetics of a Processible Binary Benzoxazine Blend

Yue Tang[1*], Henry E. Symons[1], Pierangelo Gobbo[1,2], Jeroen Sebastiaan Van Duijneveldt[1], Ian Hamerton[3], Sébastien Rochat[1,3,4]

[1]School of Chemistry, University of Bristol, Cantock's Close, Bristol, BS8 1TS, U.K.

[2]Department of Chemical and Pharmaceutical Sciences, University of Trieste, Via Giorgieri 1, Trieste, 34127, Italy.

[3]Bristol Composites Institute, School of Civil, Aerospace, and Design Engineering, University of Bristol, Queen's Building, University Walk, Bristol, BS8 1TR, U.K.

[4]School of Engineering Mathematics and Technology, University of Bristol, Ada Lovelace Building, Tankard's Close, Bristol, BS8 1TW, U.K.

* Email: wendy.tang@bristol.ac.uk

Sample Formulation

Table S1. Sample formulations studied in this work, percentages are relative to the weight ratio.

System Name	Sample Name	BA-a (%)	CA-a (%)	Initiator (%)
BA-a/CA-a system	Pure BA-a	100	0	0
	85% BA-a	85	15	
	80% BA-a	80	20	
	75% BA-a	75	25	
	Pure CA-a	0	100	
Bz/I system	Pure Bz	75	25	0
	Bz 1I			1
	Bz 2I			2
	Bz 3I			3

Evolution of the viscosity of resin versus temperature

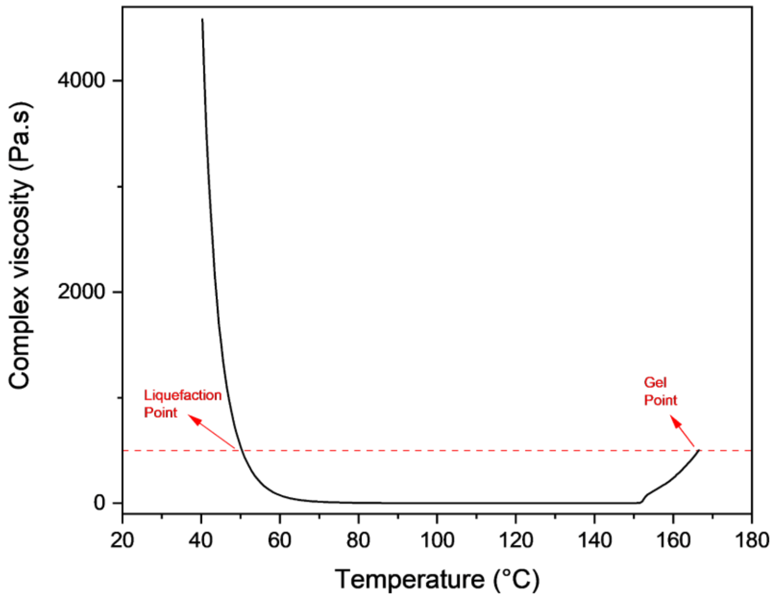

Figure S1. Schematic of the liquefaction point and gel point of a thermoset resin.

Materials

(a)

(b)

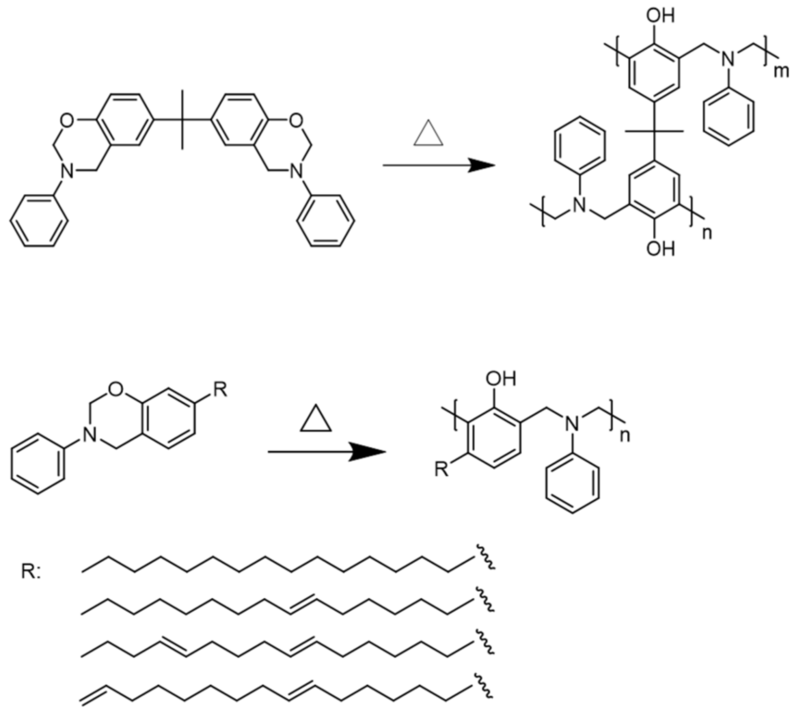

Scheme S1 Chemical structures of BA-a/CA-a systems; (a) polymerisation scheme of the BA-a benzoxazine; (b) polymerisation scheme of the CA-a benzoxazine.

Analysis of TDA Effect on Pure BA-a and Pure CA-a

The TDA initiator (2 wt%) was added separately to BA-a and CA-a, to determine if the same amount of initiator has different impacts on different benzoxazines. From DSC results (Figure S2), the polymerisation enthalpy of CA-a is increased by 37% while that of BA-a only raised by 8% with the addition of 2 wt% TDA. Moreover, the CA-a 2I formulation shows a T_p 15 °C lower compared to CA-a, while there is only 12 °C difference between the BA-a and BA-a 2I sample.

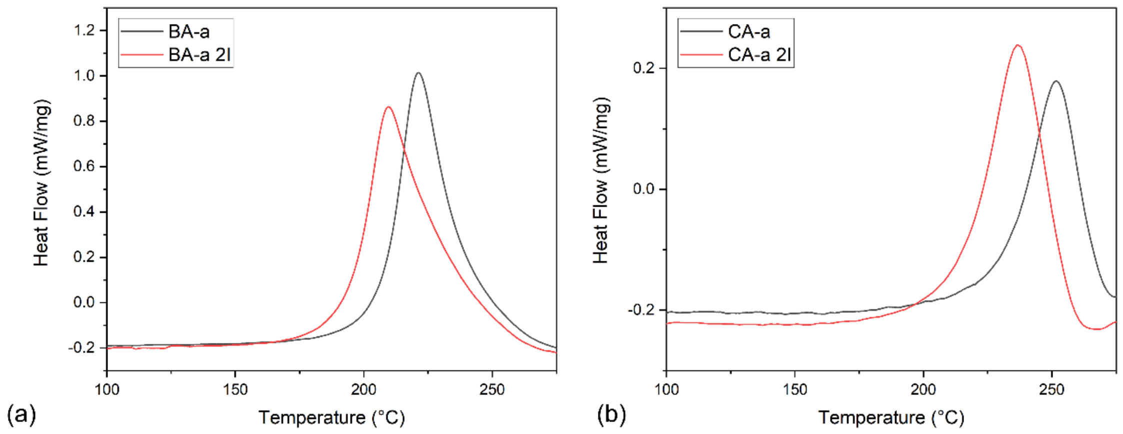

Figure S2. Scanning DSC results (plotted as heat flow, exotherm upwards, *versus* temperature) to show comparison between (a) BA-a and BA-a 2I samples (b) CA-a and CA-a 2I samples.

DMTA testing was also conducted on the cured BA-a 2I and CA-a 2I samples (Figure S3). It is perhaps counterintuitive that the tan δ peak of CA-a 2I sample is shifted to a lower temperature and shows a higher intensity with a much-increased polymerisation enthalpy. The phenomenon can be explained if another functional group in CA-a, the double bond in the long chain, is taken into consideration. The double bond might be more activated and participate into the polymerisation with the addition of the initiator, which could somehow disturb the reaction of the oxazine ring.

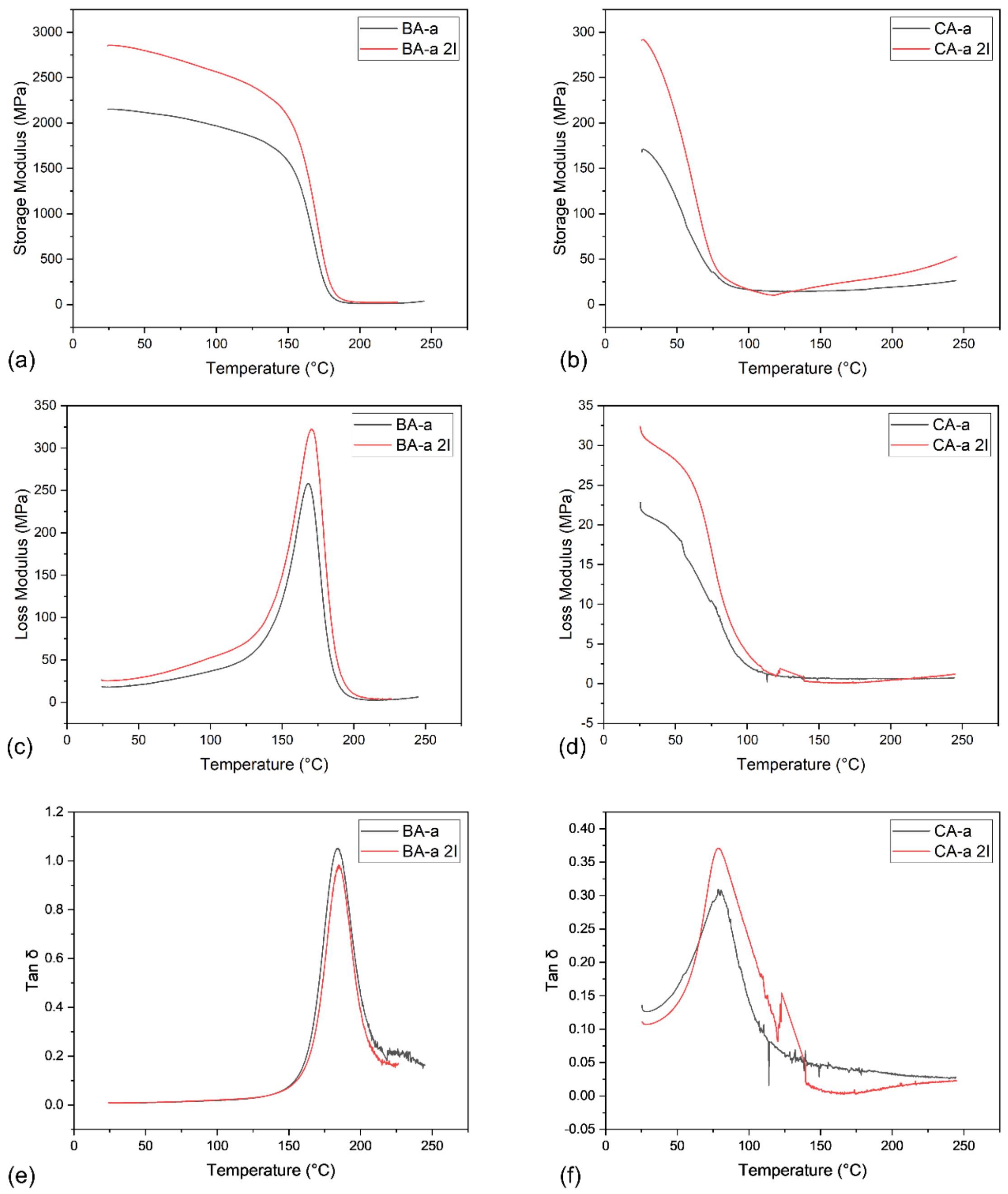

Figure S3. Scanning DMTA results of storage modulus data *versus* temperature: (a) BA-a and BA-a 2I; (b) CA-a and CA-a 2I; loss modulus data *versus* temperature: (c) BA-a and BA-a 2I; (d) CA-a and CA-a 2I; tan δ data versus temperature: (e) BA-a and BA-a 2I; (f) CA-a and CA-a 2I; measurements were performed in single cantilever mode at a frequency of 1 Hz and a strain with amplitude of 15 μm.

Rheology of TDA-incorporated Sample

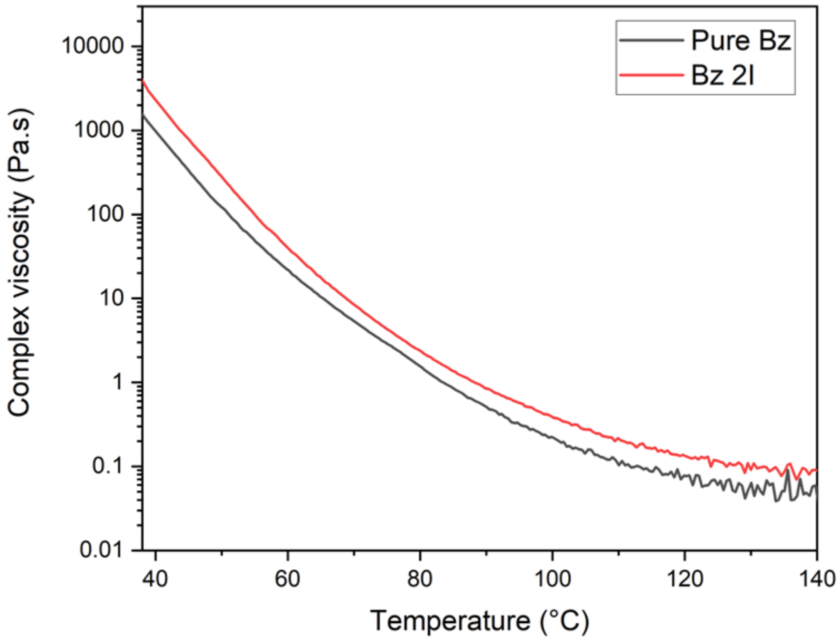

Figure S4. Plot of complex viscosity data *versus* temperature for the Bz and the Bz 2I samples.

Storage Modulus of Samples from DMTA

The storage moduli of samples at room temperature were recorded as a reference of the mechanical strength (Figure S5). As the PBz 2I and pure BA-a samples do not seem fully cured following the curing schedule (2 h at 180 °C + 1 h at 200 °C), PBz 2I was exposed to a further 1h post cure at 220 °C (Pbz 2I PC) for a more accurate storage modulus, while the storage of pure BA-a was obtained from reference.[1]

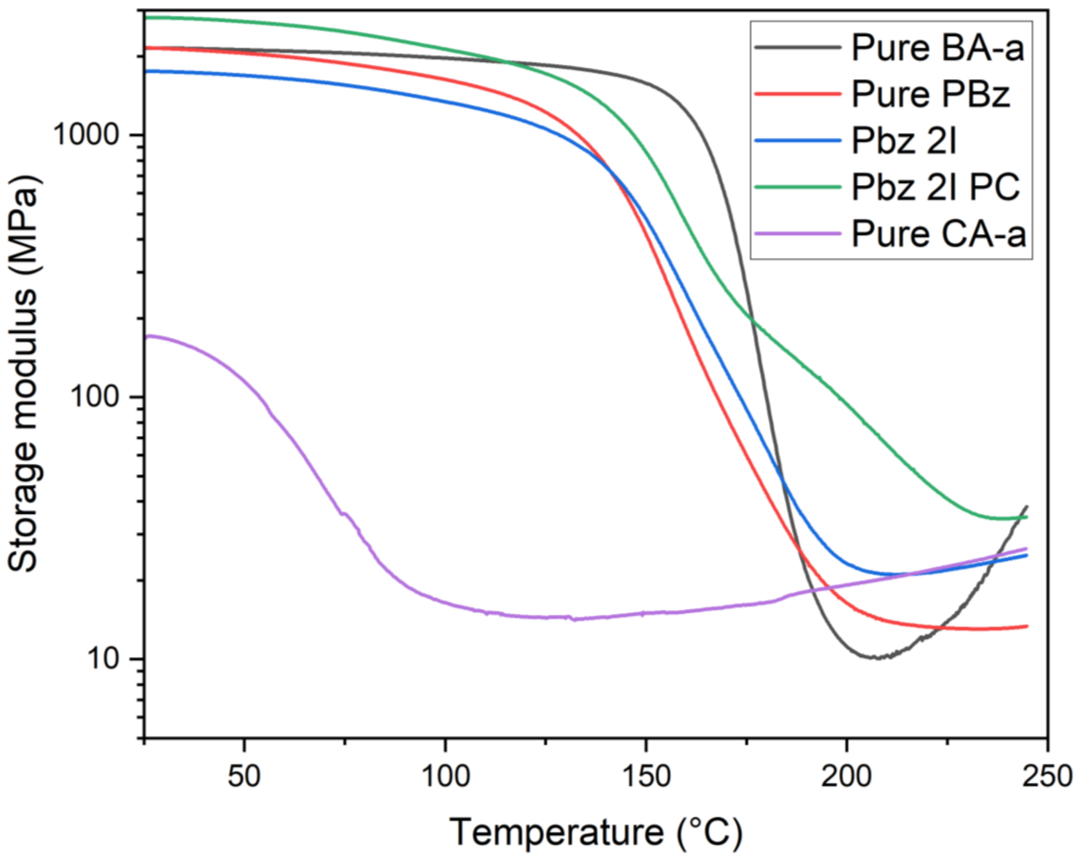

Figure S5. Plots of storage modulus data *versus* temperature obtained from DMTA testing performed on various cured PBz resins in single cantilever mode at a frequency of 1 Hz and a strain with amplitude of 15 µm.

Determination of the Cure Schedule of Bz 2I Sample

Bz 2I samples were heated rapidly to individual temperatures (160 °C, 180 °C, 200 °C, 220 °C, and 240 °C, respectively) and held for 1 hour. A DSC rescan was then performed to detect the presence of any residual polymerisation. Table S2 and Figure S7 show how the Bz 2I samples performed while being held at different temperatures. It should be noticed, during the fast-heating stage to the dwell temperature, some samples may already start curing, making it impossible to determine the enthalpies in this stage. Consequently, the isothermal ΔH values

shown here are inevitably slightly lower than the real values, especially for the samples held at higher isothermal temperatures.

The samples held over 200 °C show no obvious residual curing during the rescanning, while the specimens held at 180 °C and 160 °C show significant residual polymerisation enthalpies there. In addition, the higher temperatures result in much sharper polymerisation peaks, as well as higher reaction rates. For instance, the specimen held at 240 °C only needed around 500 s to polymerise completely, while the sample dwelling at 160 °C took more than 3000 s to achieve a degree of cure of ~0.4. The results indicate how various dwelling temperatures can change the curing process to a large extent.

Table S2. Enthapies of isothermal scanning and corresponding dynamic rescanning of Bz 2I samples.

Temperature (°C)	Isothermal ΔH		Rescan ΔH		Total ΔH	
	(J g^{-1})	(kJ mol^{-1})	(J g^{-1})	(kJ mol^{-1})	(J g^{-1})	(kJ mol^{-1})
160	124	33	54	14	178	47
180	211	56	10	3	221	59
200	253	67	-	-	253	67
220	261	69	-	-	261	69
240	276	73	-	-	276	73

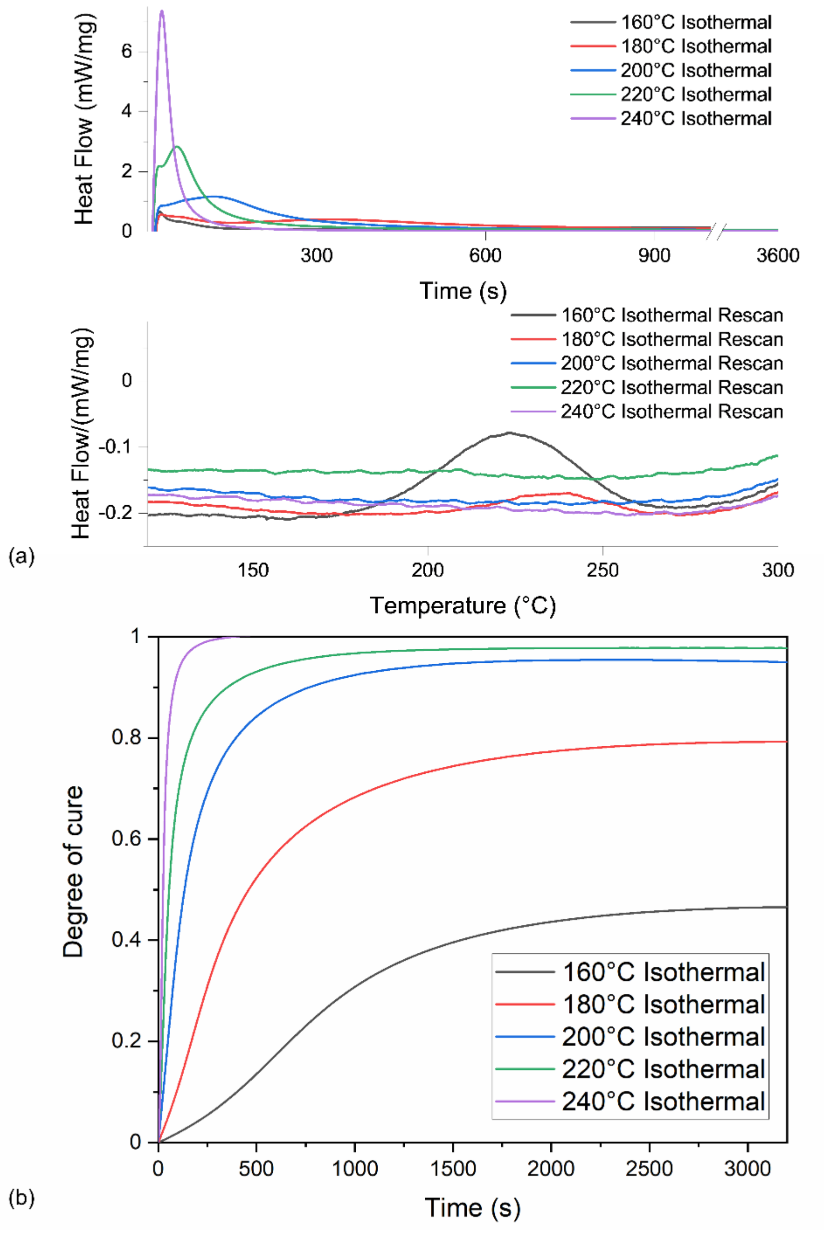

Figure S6. Thermal analysis of Bz 2I samples; (a) DSC curves showing plots of heat flow (exotherm upwards) *versus* temperature for different isothermal temperatures and corresponding rescan experiments; (b) plots of degree of cure *versus* dwell time for different isothermal temperatures.

Apparent Activation Energies

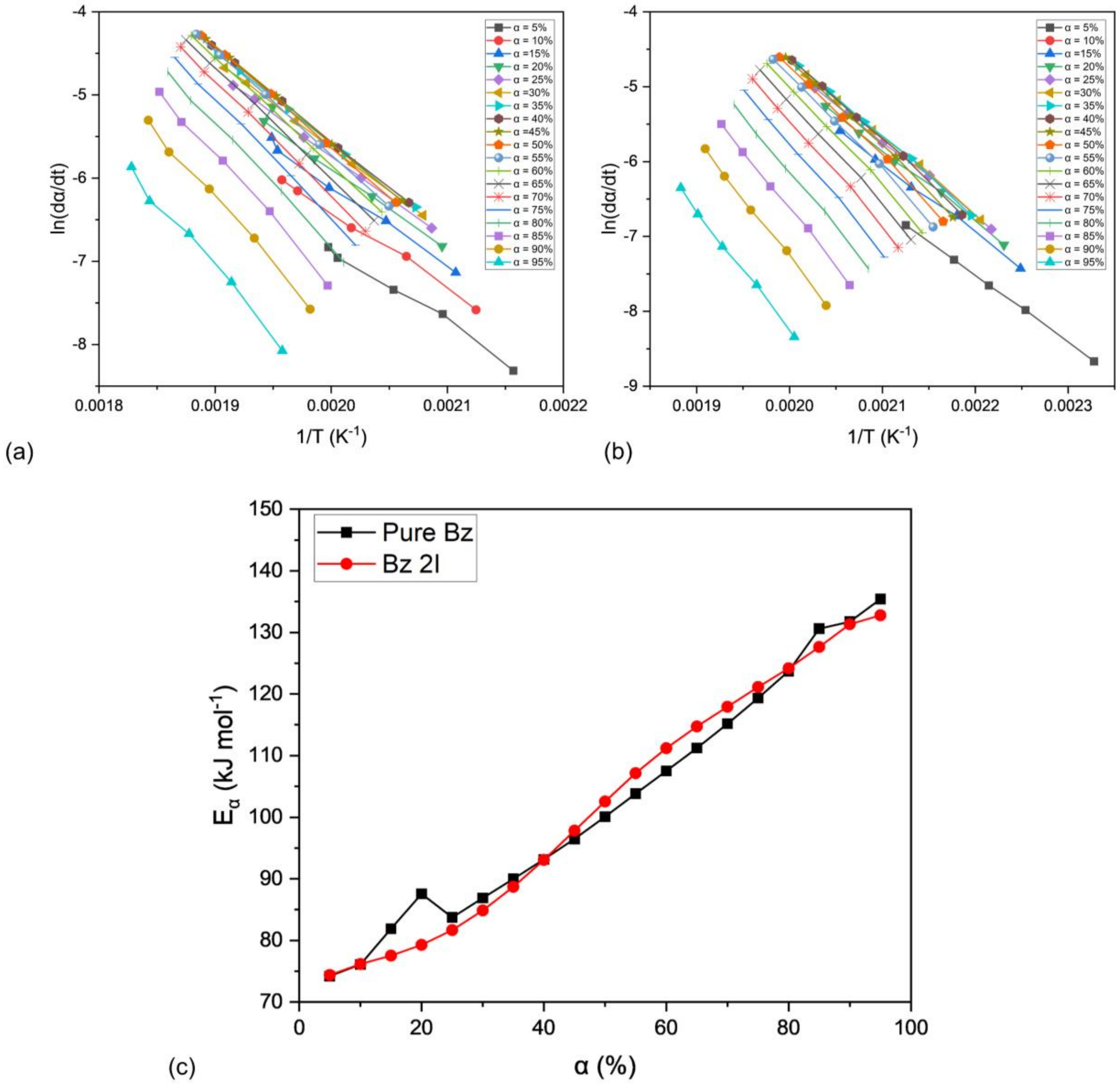

Figure S7. (a)Friedman plot of pure Bz;(b)Friedman plot of Bz 2I;(c)Values of the apparent activation energy obtained different conversions of pure Bz (■) and Bz 2I (●).

Fourier Transform Infrared (FTIR)

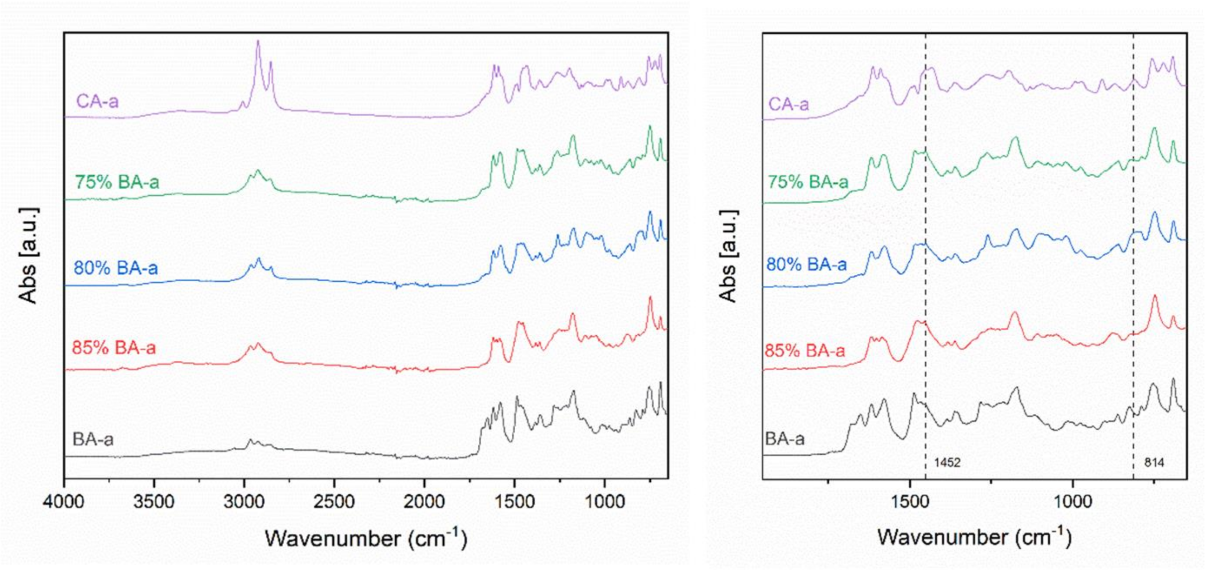

Figure S8. FTIR spectra (plotted as absorbance *versus* wavenumber) for cured polybenzoxazine resins with different formulations using PerkinElmer FT-IR Spectrometer.

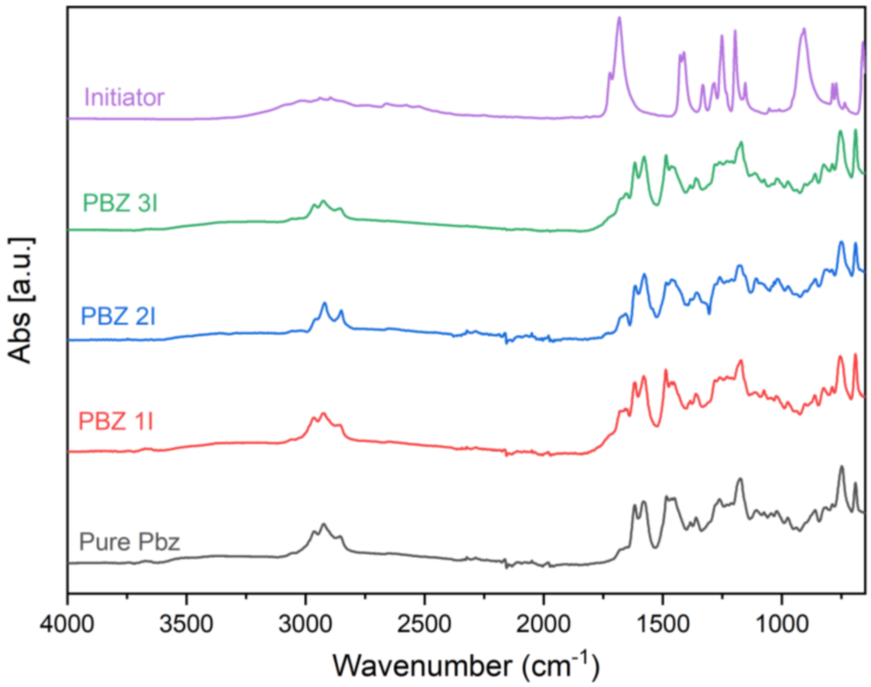

Figure S9. FTIR spectra (plotted as absorbance *versus* wavenumber) of the PBz with various amounts of initiator.

References

(1) Takeichi, T.; Kawauchi, T.; Agag, T. Polybenzoxazine/Polyimide Alloys. In Handbook of Benzoxazine Resins, Elsevier, 2011; pp 378-387.
